# Malignant uterine/broad-ligament perivascular epithelioid cell tumor (PEComa) coexisting with leiomyoma in a postmenopausal woman: A case report

**DOI:** 10.1016/j.crwh.2025.e00767

**Published:** 2025-11-15

**Authors:** Derar I.I. Ismerat, Barah K.S. Alsalameh, Majd Oweidat, Karam M. Hmidat, Areen Sulaiman, Yara Atawneh, Baraa Z. Zawahra, Izzeddin A. Bakri, Hani Hour

**Affiliations:** a College of Medicine, Hebron University, Hebron, West Bank, Palestine; b Department of Obstetrics & Gynecology, Al-Ahli Hospital, Hebron, West Bank, Palestine; c Palestine Red Crescent Specialized Hospital - Hebron, Hebron, West Bank, Palestine; d Department of Pathology, Al-Ahli Hospital, Hebron, West Bank, Palestine; e Faculty of Medicine, Al-Quds University, Jerusalem, West Bank, Palestine; f Division of Oncology, Department of Internal Medicine, Al-Ahli Hospital, Hebron, West Bank, Palestine

**Keywords:** PEComa, Uterus, Postmenopausal bleeding, Fibroid, Broad ligament, Hysterectomy

## Abstract

Perivascular epithelioid cell tumor of the uterus is a rare mesenchymal neoplasm with variable malignant potential and nonspecific clinical and radiologic features, often mistaken preoperatively for leiomyoma. This report describes the case of a postmenopausal woman with seven months of persistent postmenopausal bleeding, pelvic pain, and symptomatic anemia. Pelvic ultrasound and magnetic resonance imaging demonstrated a markedly enlarged, irregular uterus with multiple masses interpreted as fibroids. The patient underwent total abdominal hysterectomy with bilateral salpingo-oophorectomy for refractory bleeding and concern for occult malignancy. Histopathological examination revealed coexisting benign leiomyomas and a malignant perivascular epithelioid cell tumor involving the uterine wall with extension into the broad ligament. The tumor showed epithelioid morphology with brisk mitotic activity, necrosis, and dual myomelanocytic differentiation on immunohistochemistry (HMB-45 and desmin positive). Whole-body positron emission tomography demonstrated no metastatic disease. Complete resection followed by adjuvant radiotherapy was associated with resolution of bleeding and pain. This case highlights that malignant uterine perivascular epithelioid cell tumor can mimic benign leiomyoma, may coexist with a true leiomyoma, and should be considered in women with persistent postmenopausal bleeding and an enlarged fibroid-appearing uterus.

## Highlights

• Malignant uterine PEComa can mimic benign leiomyomas on imaging.
• Presented with persistent postmenopausal bleeding and large uterine masses.
• Tumor involved uterine wall and broad ligament, coexisting with leiomyoma.
• Complete excision with adjuvant radiotherapy achieved postoperative control.

## 1 Introduction

Perivascular epithelioid cell tumors (PEComas) are a rare group of mesenchymal neoplasms composed of distinctive epithelioid cells that exhibit dual myomelanocytic differentiation, typically co-expressing melanocytic markers such as HMB-45 and smooth muscle markers including desmin or actin [1]. The uterus is considered one of the frequent gynecologic sites of PEComa involvement; nevertheless, malignant uterine PEComas remain extremely rare [2].

Patients often present with nonspecific complaints such as abnormal uterine bleeding (AUB) or postmenopausal bleeding (PMB), pelvic pain, or the incidental finding of an enlarging pelvic mass [3]. Because their clinical features overlap substantially with those of benign uterine conditions, particularly leiomyomas, and preoperative imaging lacks specific features to distinguish them reliably, PEComas are rarely suspected preoperatively. As a result, diagnosis is typically made after the histopathologic assessment [4].

A central clinical challenge after establishing the diagnosis lies in prognostic stratification. Uterine PEComas encompass a spectrum ranging from indolent tumors to aggressive lesions with high recurrence and metastasis rates. Complete surgical excision is widely accepted as the primary treatment for localized disease [3,4].

To our knowledge, this is the first reported case of a postmenopausal woman with a malignant uterine PEComa extending into the broad ligament and coexisting with benign leiomyomas, presenting primarily with persistent PMB.

## 2 Case Presentation

A woman in her forties, multiparous and postmenopausal for approximately two and a half years, presented to the emergency department with a seven-month history of PMB described as intermittent episodes of light spotting punctuated by unpredictable bouts of heavier flow with passage of small clots, often requiring multiple pads over several hours and occurring without clear triggers; the bleeding was accompanied by lower abdominal pain characterized as a dull, cramping discomfort centered suprapubically, sometimes escalating to colicky waves that intensified during the heavier bleeding episodes, occasionally radiating to the lower back, and partially relieved by simple analgesics. She also reported generalized weakness and progressive fatigue consistent with chronic blood loss.

Her medical history included recently diagnosed hyperthyroidism identified incidentally several months prior, for which she was taking carbimazole 20 mg twice daily; she was clinically euthyroid at presentation. She had longstanding anemia with a hemoglobin concentration of 9 g/dL and no history of transfusion before this admission. Surgical history was limited to childhood tonsillectomy. There was no known family history of malignancy and no known drug or food allergies.

On admission, her regular medications were medroxyprogesterone acetate 5 mg twice daily and carbimazole 20 mg twice daily. She appeared mildly pale, with stable vital signs, and systemic examination was unremarkable, although pelvic examination revealed an enlarged, irregular, firm uterus. Pelvic ultrasound (US) and magnetic resonance imaging (MRI) showed a myomatous uterus with multiple leiomyomas, including three anterior intramural fibroids (largest approximately 12 × 12 cm) and a subserosal lesion measuring about 5 × 5 cm; endometrial thickness assessment was limited by cavity distortion.

Given the persistence of PMB despite medical therapy, associated symptomatic anemia, and the presence of a markedly enlarged irregular uterus in the postmenopausal setting, surgery was recommended to exclude underlying malignancy. The patient also expressed a clear preference for definitive surgical management, which together constituted the decisive factor to proceed with operative management.

**Fig. 1:**
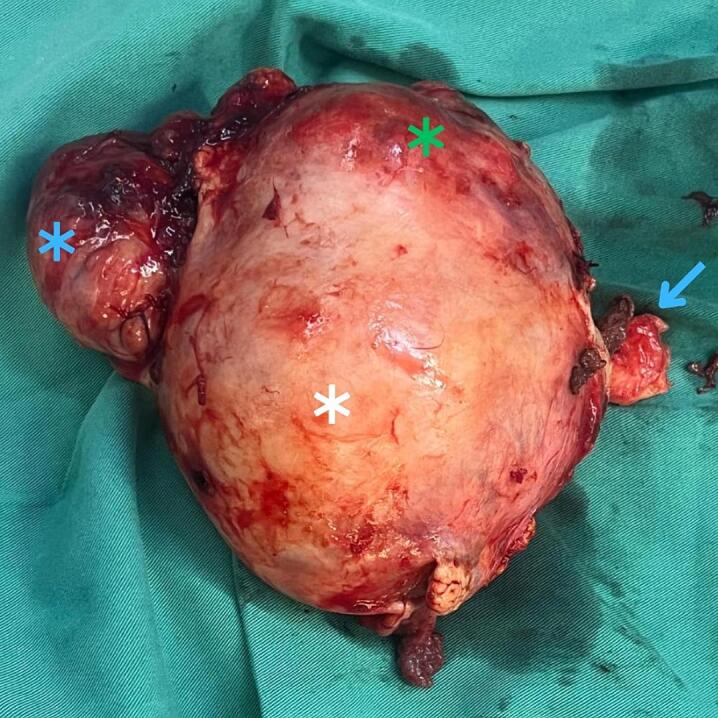
Intraoperative photograph of the hysterectomy specimen immediately after extraction. The uterus is markedly enlarged and distorted by multiple masses. The blue asterisk marks the malignant PEComa. The white asterisk marks the dominant intramural/subserosal leiomyomatous mass (fibroid). The green asterisk indicates the uterine corpus. The blue arrow identifies the cervix. (For interpretation of the references to colour in this figure legend, the reader is referred to the web version of this article.)

Routine preoperative blood grouping and cross-matching were performed. Under general anesthesia she underwent total abdominal hysterectomy with bilateral salpingo-oophorectomy (TAH-BSO); intraoperatively the adnexal anatomy was distorted by the uterine masses (Fig. 1), and two units of packed red blood cells were transfused. The surgical specimen comprised the uterus with bilateral adnexa; the uterus measured roughly 10 × 13.5 × 10 cm and contained multiple masses, including a large ill-defined intramural lesion (9.5 × 9 × 8 cm) with a heterogeneous gray, firm cut surface and necrosis compressing the endometrial cavity, a subserosal mass (6 × 5 × 5 cm), and a broad-ligament mass (2.5 × 2 × 5 cm).

**Fig. 2:**
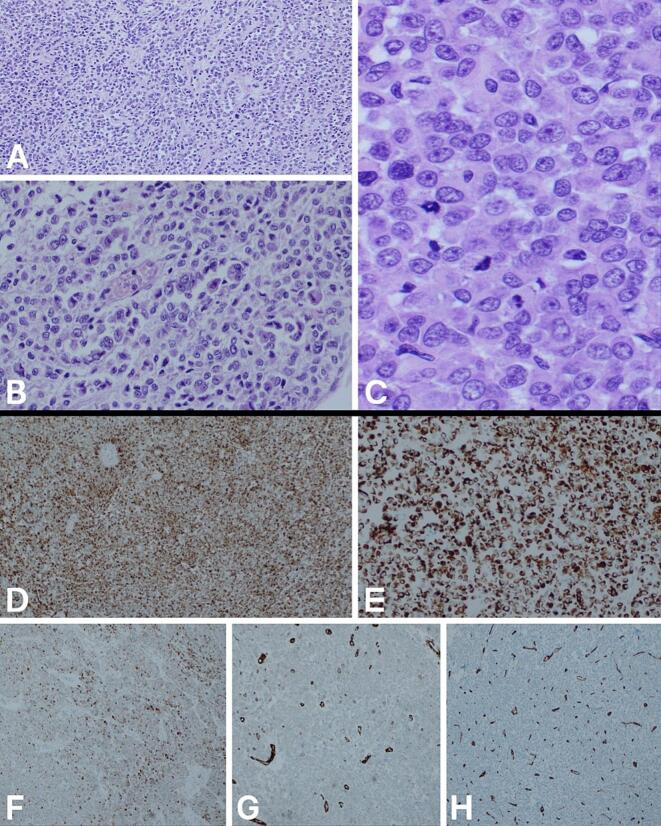
Histopathological and immunohistochemical features of the uterine tumor. (A–C) Hematoxylin and eosin-stained sections show sheets, nests, and trabeculae of non-cohesive epithelioid cells with abundant eosinophilic cytoplasm, round nuclei with vesicular chromatin, and prominent nucleoli. Delicate vascular channels are noted, and brisk mitotic activity is evident. Magnifications: A, ×10; B, ×20; C, ×40. (D—H) Immunohistochemical profile: diffuse cytoplasmic positivity for HMB-45 (D) and desmin (E); patchy positivity for pancytokeratin (F); negative staining for h-caldesmon (G) and CD34 (H).

Histopathologic examination (Fig. 2) confirmed the coexistence of benign leiomyomas and a malignant PEComa involving the uterine wall with extension into the broad ligament. The PEComa diagnosis was supported by its characteristic morphology and immunohistochemical profile, and the tumor was staged as pT2a Nx Mx. Whole-body positron emission tomography (PET) was performed to evaluate for residual disease or metastasis and showed no suspicious lesions.

After multidisciplinary discussion including gynecologic oncology, no further surgery was advised in light of negative imaging and complete resection. Given the pathologic risk features, adjuvant radiotherapy was recommended with the goal of reducing loco-regional recurrence risk. At postoperative follow-up visits the patient reported complete resolution of bleeding and pain, with improvement in energy.

## 3 Discussion

This report details a malignant uterine PEComa in a postmenopausal woman presenting with persistent PMB, refractory anemia, and multiple large uterine masses. Importantly, the PEComa coexisted with a true leiomyoma, creating the clinical impression of a uterus enlarged solely by fibroids.

The patient's presentation with PMB is notable, as this symptom is more commonly associated with endometrial cancer or atrophic changes in postmenopausal women [5]. Uterine bleeding has been the most frequently documented symptom of uterine PEComa, reported in roughly one-third of cases [4].

However, the persistence of bleeding despite medical management with progestins, coupled with significant anemia and a markedly enlarged irregular uterus, raised oncologic concern. Imaging in this case showed multiple masses interpreted as leiomyomas, which is consistent with prior reports that uterine PEComas are almost invariably labeled preoperatively as fibroids [3,4]. The diagnostic challenge was amplified here by the fact that the patient truly did have a large leiomyoma in addition to the malignant PEComa. At present, no established radiologic features reliably distinguish PEComa from leiomyoma, particularly in uteri containing both lesions.

However, histopathology remains the diagnostic cornerstone. In this case, the tumor showed epithelioid cells with eosinophilic cytoplasm arranged in nests and trabeculae, with brisk mitotic activity and necrosis, while immunohistochemistry confirmed dual myomelanocytic differentiation, with strong HMB-45 and desmin expression and patchy pancytokeratin positivity.

Prognostic stratification is central. None of the available universally accepted systems has been validated. However, the modified Folpe criteria currently show the best performance for uterine PEComa [6]. In that framework, a tumor is classified as malignant when there is necrosis or when two or more high-risk features are present (size ≥5 cm, infiltrative growth, mitotic rate > 1/50 HPF, lymphovascular invasion, marked atypia) [6]. In the present case, the tumor measured ≥5 cm and showed necrosis, therefore meeting malignant status by modified Folpe.

Management in this case involved TAH-BSO, consistent with current practice where complete surgical resection remains the cornerstone for localized disease [4]. The decision to administer adjuvant radiotherapy was based on the tumor's malignant histology and involvement of the broad ligament, with the aim of reducing locoregional recurrence. The literature, however, provides no consensus on adjuvant therapy, with chemotherapy, radiotherapy, or both employed inconsistently and without clear evidence of survival benefit [3]. mTOR inhibitors have shown promise in advanced disease and represent a rational targeted approach, but their role in the adjuvant setting remains undefined [7].

According to the literature, recurrence followed primary therapy in 34.7 % of cases (median 9.5 months). Recurrences most often involved lungs (60 %), pelvis (31.4 %), liver (17.1 %), and bone/kidney (each 5.7 %). Metastatic disease at diagnosis was 8.9 %, chiefly in the lungs (55.6 %) and pelvic nodes (33.3 %); overall mortality was 14.9 % [4].

This report adds to the limited literature on postmenopausal uterine PEComa and is among the few documenting direct broad-ligament involvement. To the authors' knowledge, this is the first report of a malignant uterine and broad ligament PEComa in a postmenopausal woman that simultaneously coexisted with histologically confirmed benign leiomyomas and presented primarily as persistent PMB. This constellation underscores that persistent PMB in the setting of an apparently “fibroid” uterus should prompt consideration of rare malignancies beyond the more common endometrial or uterine sarcomatous etiologies, and further highlights the limitations of imaging in distinguishing benign leiomyomas from malignant PEComa.

## 4 Conclusion

In conclusion, malignant uterine PEComa with broad-ligament extension is rare. It may be misdiagnosed as a benign leiomyoma preoperatively, and may even coexist with a true leiomyoma, further obscuring the true pathology. Definitive diagnosis requires histopathologic evaluation. Complete surgical resection remains the mainstay of treatment, and risk stratification guides consideration of adjuvant therapy.

## Contributors

Derar I.I. Ismerat contributed to patient care, acquiring the data and drafting the manuscript.

Barah K. S. Alsalameh contributed to patient care, acquiring the data and drafting the manuscript.

Majd Oweidat contributed to patient care, conception of the case report and revising the article critically for important intellectual content.

Karam M Hmidat contributed to acquiring the data and drafting the manuscript.

Areen Sulaiman contributed to acquiring the data and drafting the manuscript.

Yara Atawneh contributed to acquiring the data and drafting the manuscript.

Baraa Z. Zawahra contributed to patient care, acquiring the data and drafting the manuscript.

Izzeddin A. Bakri contributed to patient care, acquiring the data and drafting the manuscript.

Hani Hour contributed to patient care, acquiring the data and drafting the manuscript.

All authors approved the final submitted manuscript.

## Patient consent

Written informed consent was obtained from the patient for publication of this case report and accompanying images.

## Provenance and peer review

This article was not commissioned and was peer reviewed.

## Funding

No funding from an external source supported the publication of this case report.

## Declaration of competing interest

The authors declare that they have no competing interest regarding the publication of this case report.
